# Sustained Liver Glucose Release in Response to Adrenaline Can Improve Hypoglycaemic Episodes in Rats under Food Restriction Subjected to Acute Exercise

**DOI:** 10.1155/2014/969137

**Published:** 2014-02-27

**Authors:** Lucas K. R. Babata, Maria M. D. Pedrosa, Rosângela F. Garcia, Márcia V. Peicher, Vilma Aparecida Ferreira de Godoi

**Affiliations:** ^1^State University of Maringá, 87020900 Maringá, PR, Brazil; ^2^Department of Physiological Sciences (DFS), State University of Maringá (UEM), Avenida Colombo 5790, 87020900 Maringá, PR, Brazil

## Abstract

*Background*. As the liver is important for blood glucose regulation, this study aimed at relating liver glucose release stimulated by glucagon and adrenaline to *in vivo* episodes of hypoglycaemia. *Methods*. The blood glucose profile during an episode of insulin-induced hypoglycaemia in exercised and nonexercised male Wistar control (GC) and food-restricted (GR, 50%) rats and liver glucose release stimulated by glucagon and adrenaline were investigated. *Results*. In the GR, the hypoglycaemic episodes showed severe decreases in blood glucose, persistent hypoglycaemia, and less complete glycaemic recovery. An exercise session prior to the episode of hypoglycaemia raised the basal blood glucose, reduced the magnitude of the hypoglycaemia, and improved the recovery of blood glucose. In fed animals of both groups, liver glucose release was activated by glucagon and adrenaline. In fasted GR rats, liver glycogenolysis activated by glucagon was impaired, despite a significant basal glycogenolysis, while an adrenaline-stimulated liver glucose release was recorded. *Conclusions*. The lack of liver response to glucagon in the GR rats could be partially responsible for the more severe episodes of hypoglycaemia observed *in vivo* in nonexercised animals. The preserved liver response to adrenaline can partially account for the less severe hypoglycaemia in the food-restricted animals after acute exercise.

## 1. Introduction

The liver is a central organ for the regulation of energy homeostasis in general and blood glucose homeostasis in particular. It responds directly and autonomously to blood glucose concentration [1, 2] and is as well a central target of the neural and hormonal factors controlling energy metabolism; while insulin promotes glycogen synthesis and glycolysis in the liver, the counterregulatory hormones—glucagon, adrenaline, cortisol, and growth hormone—are glycogenolytic and gluconeogenic [3, 4]. Together with their extrahepatic actions, the counterregulatory hormones tend to increase the blood glucose level, while the actions of insulin on the liver and other tissues are hypoglycaemic.

When glucose drops below the physiologically acceptable range, the counterregulatory system increases the production/release of glucose to be used, primarily by the brain, and supplies alternative energy substrates for the other tissues [3]. In this scenario, glucagon, because of its fast-acting effects on the liver, represents the first line of defence against hypoglycaemia [4].

During exercise, liver release of glucose helps to keep blood glucose within normal limits. This comes from glycogenolysis when glycogen stores are available [5, 6], especially during short-term moderate- to high-intensity exercises [2]. Although exercise activates many counterregulatory agents [7, 8], the increased blood levels of catecholamines deserve mention [7, 9, 10].

In adult rats raised under food restriction, hypoglycaemic episodes are characterized by severe decrease in blood glucose levels, persistent hypoglycaemia, and less complete glycaemic recovery [11], probably as a result of increased insulin sensitivity and impaired activation of the counterregulatory hormones [12–14]. However, it was observed that acute exercise has a positive effect on this hypoglycaemic profile: an exercise session previous to an episode of hypoglycaemia in these animals raised the basal blood glucose level, reduced the rate of decrease and the magnitude of the hypoglycaemia, and improved the recovery of the normal blood glucose level [15].

As the liver is an important metabolic centre for the regulation of blood glucose, we hypothesized that the counterregulation of hypoglycaemia and its hepatic effects, which seem to be compromised in animals under food restriction, could be potentiated by exercise, giving rise to less severe hypoglycaemic profiles in exercised compared to nonexercised animals [15]. Therefore, we reevaluated the blood glucose profile during an episode of insulin-induced hypoglycaemia (IIH) in exercised and nonexercised rats and investigated the liver glucose release stimulated by glucagon and adrenaline, hormones with hyperglycaemic actions on the liver that are relevant during hypoglycaemia and exercise. Our purpose was to correlate the liver glucose release stimulated by glucagon and adrenaline with the profiles of IIH recorded *in vivo*.

## 2. Materials and Methods

### 2.1. Experimental Groups

The Wistar dams and their puppies were kept in the animal house of the Department of Physiological Sciences under controlled light : dark cycles (12 h L : 12 h D) and temperature (22 ± 2°C). All the procedures were approved by the Commission on Ethics in Animal Experimentation of the University and are in accordance with EU Directive 2010/63/EU for animal experiments.

Newborn litters were organized so that each dam had either six or 12 puppies (control group: GC; restriction group: GR, resp.). The mothers had free access to water and chow (Nuvilab CR1; Nuvital, Curitiba, PR, Brazil) during gestation and lactation.

After weaning, at the age of 21 days, the puppies were kept in plastic boxes in groups of four animals. The GC rats had a free supply of water and chow, while those in the GR were subjected to a food restriction of 50% [11, 16, 17] until the age of 50–60 days, when the experiments were carried out. Only males were used in this investigation.

### 2.2. Insulin-Induced Hypoglycaemia (IIH): *In Vivo* Experiments

For the IIH experiments, preceded or not by exercise, the animals were fasted overnight (approx. 14 hours). The next morning they were injected i.p. with regular insulin (1 U·kg^−1^ b.w.; Novolin; Novo Nordisk, Montes Claros, MG, Brazil) [11]. Blood samples were collected from the tail vein at 0, 15, 30, 60, 120, 180, 240, and 300 min, time 0 being the moment immediately before the insulin injection. Blood glucose was determined with a glucometer (Optium Xceed, Abbott, Witney, UK).

### 2.3. Session of Acute Exercise: *In Vivo* Experiments

Some of the rats from both groups were subjected to exercise before IIH (groups GCex and GRex). The exercise session consisted of a run in an adapted programmed treadmill (KT3000, Inbramed, Porto Alegre, RS, Brazil). The treadmill speed increased 0.2 km·h^−1^ every two minutes from an initial speed of 0.4 km·h^−1^ and continued until exhaustion. This was established as the moment when the animals could not keep up with the treadmill speed or stopped running completely, characterizing an acute session of exercise.

### 2.4. Liver Glucose Release: *In Situ* Liver Perfusion

For the experiments of *in situ* liver perfusion, the rats, either fed or under overnight fasting, were anesthetized by i.p. injection of sodium thiopental (40 mg·kg^−1^ b.w.) and had both the portal vein and inferior cava vein cannulated. The liver was perfused with Krebs-Henseleit buffer (KH, pH 7.4, 37°C) in a nonrecirculating system. The perfusion fluid was pumped through a membrane oxygenator saturated with O_2_/CO_2_ (95/5%) before entering the liver through the portal vein. Immediately after the beginning of the perfusion, the diaphragm was opened and the thoracic portion of the inferior cava vein was ligated [11, 17].

After 30 min of stabilization, during which the liver was perfused with KH, samples of the effluent fluid were collected through the inferior cava vein each 5 min. During the period of collection the liver was perfused for 10 min with KH (basal perfusion) and then with KH containing glucagon (1 nM for 20 min; Glucagen, Novo Nordisk, Montes Claros, MG, Brazil) or adrenaline (1 *μ*M for 30 min; Adren, Hipolabor, Belo Horizonte, MG, Brazil) (stimulated perfusion) [18, 19]. Some of the perfusion experiments with adrenaline were carried out in overnight fasted GC or GR animals immediately after an acute session of exercise.

**Figure 1:**
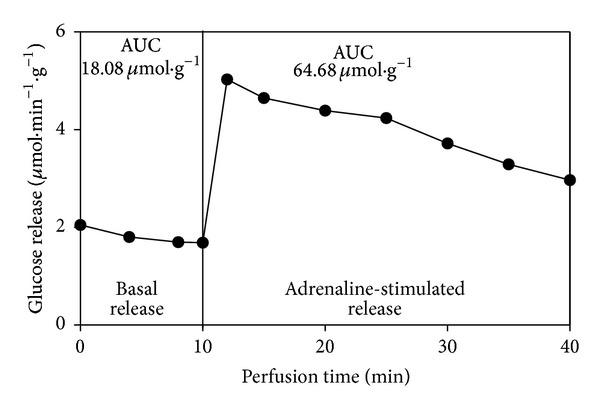
Illustrative graph of liver glucose release in a fed control rat, before (basal release) and during (stimulated release) perfusion with adrenaline (1 *μ*M). The vertical line indicates the transition from basal perfusion (KH only) to stimulated perfusion (KH with adrenaline 1 *μ*M). The AUC of basal glucose release considered all glucose released during the 10-min interval. AUC of stimulated release considered those glucose values above the basal release during the 30-min interval.

The concentrations of glucose [20], lactate [21], and pyruvate [22] in the samples were determined spectrophotometrically and calculated as *μ*mol·min^−1^·g^−1^ liver. Based on the values obtained during basal and stimulated perfusion, the area under the curve (AUC) of the liver release of each compound was calculated and expressed as *μ*mol·g^−1^ liver.  Figure 1 illustrates liver glucose release during basal and stimulated perfusion with adrenaline in a fed control rat. The AUCs in this investigation were calculated from similar experiments.

The means of the AUCs of lactate and pyruvate in the adrenaline-stimulated perfusion were used to calculate the rates of glycolysis (= pyruvate + lactate) and glycogenolysis [= glucose + 1/2 (pyruvate + lactate)] of the groups [23].

### 2.5. Statistical Analysis

The data were treated statistically using the nonpaired Student's *t*-test. Values of *P* < 0.05 were considered significant. The statistical analyses were carried out using Prism 5.01 (GraphPad software, San Diego, CA, USA). The data are shown as mean ± standard deviation (SD).

## 3. Results

### 3.1. Insulin-Induced Hypoglycaemia

**Figure 2:**
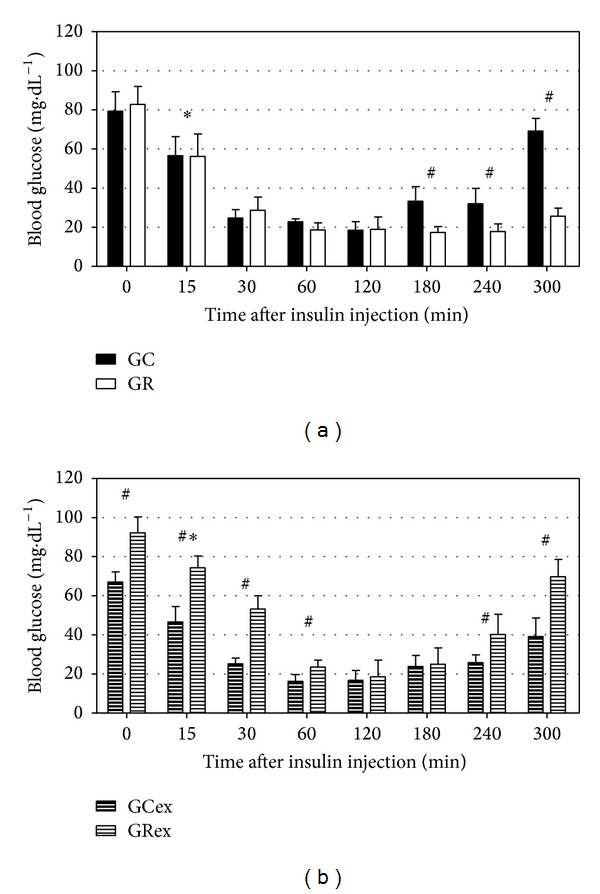
Blood glucose profiles of control and food-restricted rats during insulin-induced hypoglycaemia, either without exercise ((a), groups GC and GR) or after an acute exercise session ((b), groups GCex and GRex). Insulin was i.p. injected at time 0 min and blood glucose was recorded for 300 min. Mean ± SD of 6–12 observations at each time. *P < 0.01 compared with time 0 min of the same group; ^#^ *P* < 0.01 between groups at the same time, according to Student's *t*-test.

Figure 2 shows blood glucose profiles after overnight fasting of GC and GR rats during an episode of IIH preceded by rest Figure 2(a) or acute exercise Figure 2(b). The statistical analysis showed that the basal blood glucose (time 0 min) in the GC and GR did not differ in the nonexercised animals (*P* > 0.05, Figure 2(a)) but was significantly higher in the GRex than in the GCex (*P* < 0.01, Figure 2(b)). This is because basal blood glucose was reduced in the exercised control animals (79.31 ± 9.98 mg·dL^−1^ in the GC versus  67.08 ± 5.12 mg·dL^−1^ in the GCex, *P* < 0.05), while it was increased in the exercised food-restricted group (82.84 ± 9.21 mg·dL^−1^ in the GR versus  92.21 ± 8.09 mg·dL^−1^ in the GRex, *P* < 0.05) compared with their respective nonexercised groups.

In the nonexercised animals (GC and GR, Figure 2(a)), hypoglycaemia was already present 15 min after insulin injection and its lowest values were recorded at 60 and 120 min. Blood glucose in the two groups did not differ during the first two hours (*P* > 0.05). However, blood glucose started to rise in the GC at 180 min, while the GR showed only a slight recovery at 300 min. Thus, blood glucose in the GR remained significantly lower than that in the GC (*P* < 0.01) 180 min after the injection of insulin.

After the exercise session (GCex and GRex, Figure 2(b)), insulin injection also caused a significant drop in blood glucose that continued from 15 to 60 min, in both groups. The relative decrease in blood glucose was of the same magnitude in both exercised groups; however, the GRex showed higher absolute blood glucose values during the first 60 min of the IIH, so that the hypoglycaemic profile was markedly attenuated in the GRex compared with the GCex. The glycaemic recovery was significantly better in the GRex than in the GCex at 240 and 300 min (*P* < 0.01).

### 3.2. Liver Glucose Release: Fed Animals

In the fed state, the basal release of glucose (i.e., before perfusion with glucagon or adrenaline) was high both in the GC and the GR. The perfusion with glucagon or adrenaline caused an additional, immediate, and sustained increase in liver glucose release.

The AUCs for the basal and stimulated perfusion with glucagon or adrenaline in the fed GC and GR animals are shown in Table 1. Glucagon increased liver glucose release in both groups, but in a more significant and sustained way in the GR compared with the GC (*P* < 0.01 in the GR versus  *P* < 0.05 in the GC in comparison with the respective basal perfusion). The liver glucose release stimulated by adrenaline was markedly higher than the basal release in the fed GC and GR animals (*P* < 0.01) but was not different between the groups (*P* > 0.05).

### 3.3. Liver Glucose Release: Overnight Fasted Animals

After fasting and without a previous exercise session, the animals of the GR showed a high basal glucose release (Table 2), as observed in the fed state (Table 1); on the other hand, the basal glucose release of the GC was very low, so that basal glucose release after overnight fasting differed significantly between GC and GR (*P* < 0.01). In the overnight fasted GR, adrenaline significantly increased liver glucose release, but not in the fasted GC (Table 2). None of the fasted groups responded to the perfusion with glucagon.

Liver glucose release after the exercise session was increased by adrenaline in both groups (*P* < 0.01, Table 3). However, the values obtained for the exercised GR animals (Table 3) were much lower than those observed in the nonexercised GR (Table 2), both for the basal and stimulated perfusion. On the other hand, the basal and stimulated values did not differ statistically between the exercised (Table 3) and nonexercised animals (Table 2) in the GC.

Table 4 shows the liver release of pyruvate and lactate during the perfusion with adrenaline in the fasted GC and GR animals either subjected or not to an acute exercise session. Under adrenaline stimulation, the liver release of pyruvate was lower and that of lactate was higher in the GR than in the GC (*P* < 0.05). There were no differences in the liver release of either pyruvate or lactate between the exercised groups (*P* > 0.05, Table 4).

The calculated rates of glycolysis and glycogenolysis in the presence of adrenaline are shown in Table 5. The rate of glycolysis in the nonexercised animals was higher in the GR than in the GC, but it was similar between the groups that were exercised before the liver perfusion. The GR showed higher rates of glycogenolysis than the GC in both procedures (i.e., with or without exercise before the liver perfusion with adrenaline). However, there was a marked reduction in the rate of glycogenolysis in the exercised GR compared with the nonexercised GR.

In summary, the *in vivo* IIH experiments showed a worsened glycaemic recovery in the nonexercised GR rats (Figure 2(a)); on the other hand, the profile of the hypoglycaemia was attenuated and the blood glucose recovery was improved in the GR after an acute session of exercise (Figure 2(b)). Glucagon increased the liver glucose release only in fed animals (Table 1) but had no effect in fasted rats (Table 2). The liver response to the perfusion with adrenaline in the fasted GR (Table 2) was similar to that of the fed GR (Table 1), while the liver response to adrenaline in the fasted GC (Table 2) was insignificant. After the acute exercise session, a response to adrenaline was recorded in both groups, although it was more marked in the GR (Table 3). The liver glucose release in the exercised GR, both during the basal and the stimulated perfusion (Table 3), was significantly less than that recorded in the nonexercised GR animals (Table 2). In the absence of exercise, the fasted GR showed higher rates of glycogenolysis and glycolysis (Table 5) and higher liver release of lactate (Table 4). The acute session of exercise made these parameters equal between the GC and the GR during both the basal and the adrenaline-stimulated liver perfusion.

## 4. Discussion

This work aimed at investigating whether liver glucose release and its stimulation by adrenaline and glucagon (Table 2) could explain the change in the profile of the insulin-induced hypoglycaemia observed *in vivo* in food-restricted rats subjected to an acute exercise session (Figure 2(b)).

The *in vivo* experiments in this investigation reinforce the observations previously made [15]: first, that nonexercised food-restricted animals have episodes of hypoglycaemia that are severe and show incomplete recovery and, second, that the exercise session until exhaustion increases the basal blood glucose, decreases the severity of the hypoglycaemia, and improves the glycaemic recovery [5–7, 9, 11, 15]. A heightened sensitivity to insulin and an impaired counterregulation [11, 13, 14] were suggested as possible agents behind the observations in the nonexercised animals; improvement in the actions of some counterregulatory hormones by exercise was suggested as an explanation for the latter instance.

The fall in blood glucose after the exercise session in the GCex and its rise in the GRex (Figure 2(b)) can be partially accounted for by the liver content of glycogen: the presence of glycogen in the liver is related to postexercise hyperglycaemia, while its absence leads to hypoglycaemia [24]. In this study, the fasted GC animals showed low glycogenolysis (assessed by the basal liver glucose release, Table 2), while those of the GR had a basal liver glucose release in the fasted state (Table 2) as high as in the fed state (Table 1).

The fact that all the fed animals responded to glucagon and adrenaline with a marked increase in liver glucose release indicates that the responsiveness of the hepatocytes to these hormones was not impaired. In addition, the high basal liver glucose release in the fed groups indicates the presence of glycogen. Both observations are expected during the postprandial period [19, 24, 25].

The high basal liver glucose release in the fasted GR has already been observed [11, 13, 17] and suggests that the nutritional condition of these animals (reduced supply of food since birth) changes the turnover of liver glycogen so that glycogenolysis can be detected even after many hours of fasting [19]. The rates of glycolysis and glycogenolysis in the GR during the basal perfusion (Table 5) are in agreement with those arguments. As the animals were given food at random time intervals, the more persistent storage of glycogen cannot be attributed to metabolic programming [26].

During a hypoglycaemic episode, glucagon is one of the first hyperglycaemic agents [4]. In the fasted nonexercised GR animals, the absent response of the liver to the perfusion with glucagon (Table 2), despite the marked basal glycogenolysis, could partially explain the impaired glycaemic recovery observed in the *in vivo* experiments (Figure 2(a)). The fasted GC animals did not respond to the perfusion with glucagon either (Table 2); however, it is possible that, in these freely fed animals, having significant fat stores and well-developed skeletal muscle mass [16], extrahepatic counterregulatory actions, such as greater lipid mobilization and oxidation of fatty acids, may contribute to their improved glycaemic recovery. On the other hand, the GR, with reduced adipose tissue [8, 16], would be more dependent on liver glucose release to counteract a hypoglycaemic challenge.

The liver of the fasted GR increased glucose release in response to adrenaline in a fashion very similar to that observed in the fed animals (Tables 2 and 1, resp.). As liver glycogen is an important cause of postexercise hyperglycaemia [24], it can be hypothesized that, *in vivo*, the adrenaline released by exercise would be capable of accentuating liver glycogenolysis, resulting in higher postexercise glycaemia and better glycaemic recovery during the hypoglycaemic episode in the animals under food restriction. Consistent with this, both the basal and adrenaline-stimulated liver glucose release by the exercised GR were markedly lower than those of the nonexercised GR rats. In addition, the nonexercised GR showed both a high rate of glycolysis and a high liver release of lactate. Taken together, these results suggest a considerable decrease in liver glycogen caused by the previous strenuous exercise. More than that, the rates of glycogenolysis in the GR were higher than those in the GC regardless of the exercise but were much less in the previously exercised GR compared with the nonexercised GR animals.

## 5. Conclusion

In rats under food restriction since birth, liver glycogenolysis stimulated by glucagon was impaired. This could be partially responsible for the impaired glycaemic recovery observed during *in vivo *hypoglycaemia. On the other hand, the adrenaline-stimulated liver glycogenolysis was preserved and can partially account for the improved glycaemic profile observed during hypoglycaemia in the food-restricted animals after an acute session of exercise. The results are relevant to individuals under food restriction in the sense that exercise could be beneficial to counteract occasional episodes of hypoglycaemia.

## Figures and Tables

**Table 1:** AUCs for the liver release of glucose in the fed state.

	Glucagon 1 nM		Adrenaline 1 µM	
	GC	GR	GC	GR
Basal perfusion (*μ*mol·g^−1^)	6.01 ± 1.85	10.38 ± 2.56	26.24 ± 9.28	24.39 ± 6.18
Stimulated perfusion (*μ*mol·g^−1^)	10.14 ± 3.46*	24.10 ± 2.24^#§^	64.78 ± 0.93^@^	59.6 ± 12.05^@^

Data shown as mean ± SD of 4–10 observations.

*P < 0.05 compared with the basal perfusion in the GC; ^#^ *P* < 0.01 compared with the basal perfusion in the GR; ^§^ *P* < 0.01 compared with the stimulated perfusion in the GC; ^@^ *P* < 0.01 compared with the basal perfusion in the respective group.

**Table 2:** AUCs for the liver release of glucose in the fasted state.

	Glucagon 1 nM		Adrenaline 1 µM	
	GC	GR	GC	GR
Basal perfusion (*μ*mol·g^−1^)	1.86 ± 0.71	13.50 ± 3.57*	0.82 ± 0.40	24.23 ± 4.92*
Stimulated perfusion (*μ*mol·g^−1^)	0.27 ± 0.02*	0^@^	0.92 ± 0.71	62.99 ± 13.09^#@^

Data shown as mean ± SD of 4–6 observations.

*P < 0.01 compared with the basal perfusion in the GC; ^#^ *P* < 0.01 compared with the stimulated perfusion in the GC; ^@^ *P* < 0.05 compared with the basal perfusion in the GR.

**Table 3:** AUCs for the liver release of glucose in the fasted state after an acute exercise session.

	GC	GR
Basal perfusion (*μ*mol·g^−1^)	0.67 ± 0.21	1.35 ± 0.25^#^
Adrenaline-stimulated perfusion (*μ*mol·g^−1^)	1.46 ± 0.21*	2.67 ± 0.37^∗#^

Data shown as mean ± SD of 4–7 observations.

*P < 0.01 compared with the basal perfusion in the same group; ^#^ *P* < 0.05 compared with the corresponding period in the GC.

**Table 4:** AUCs for the liver release of pyruvate and lactate.

Perfusion procedure	Pyruvate (*μ*mol·g^−1^)		Lactate (*μ*mol·g^−1^)	
	GC	GR	GC	GR
Adrenaline 1 *μ*M	0.039 ± 0.02	0*	0.654 ± 0.30	1.269 ± 0.44*
Exercise + Adrenaline 1 *μ*M	0.131 ± 0.07	0.138 ± 0.03	2.366 ± 0.46	2.027 ± 0.87

Data shown as mean ± SD of 4–8 observations.

*P < 0.05 compared with the GC with the same procedure.

**Table 5:** Rates of glycolysis and glycogenolysis*.

		Perfusion procedure	
		Adrenaline 1 *μ*M	Exercise + Adrenaline 1 *μ*M
Rate of glycolysis (*μ*mol·g^−1^)	GCb	0.72	1.57
	GRb	10.93	1.99
	GCst	0.70	2.50
	GRst	1.27	2.17

Rate of glycogenolysis (*μ*mol·g^−1^)	GCb	1.18	1.46
	GRb	29.69	2.34
	GCst	1.58	2.71
	GRst	63.62	3.75

*Calculated as described in Section 2.4.
